# Dataset on the reductive amination of phenolics with cyclohexylamine over Rh/C and Pd/C: Catalysts characterization and reaction performance

**DOI:** 10.1016/j.dib.2022.108620

**Published:** 2022-09-20

**Authors:** Maray Ortega, Benjamin L. Garrido-Lara, Raydel Manrique, Luis E. Arteaga-Pérez

**Affiliations:** a Laboratory of Thermal and Catalytic Processes (LPTC-UBB), Wood Engineering Department, University of Bio-Bio, Concepción, Chile; b Universidad de Concepción, Unidad de Desarrollo Tecnológico, UDT, Coronel, Chile

**Keywords:** Amination, Phenolics, Catalyst, Pd/C, Rh/C, Dataset

## Abstract

Secondary amines play a very important role in today's chemical industry owing to their extensive applications in agricultural, pharmaceutical, textile, polymer and in personal care fields [1] Unfortunately, most of the amine synthesis processes at the industrial level are fossil-based and imply economic and environmental problems. However, the heterogeneously catalyzed reductive amination of lignin-derived phenolics has been recognized as an efficient and ecofriendly method for the synthesis of primary or higher order amines [2]. In this sense, metal-supported catalysts, specifically palladium, and rhodium-based materials, have demonstrated their effectivity to produce secondary amines [3,4]. Therefore, there is a crescent interest in evaluating their roles within the reaction mechanisms by testing different reaction conditions and phenolics sources.

Nevertheless, there is a lack of experimental data allowing to establish a correlation between the nature of the metallic clusters, the operational parameters, and steric effects of alkyl-phenolics with the activity and selectivity to amines.

Accordingly, this dataset includes reliable experimental measurements on the use of Pd/C and Rh/C as catalysts for the reductive amination of phenols (RAPhs). A complete set of characterization techniques was applied to inspect the structural and textural properties of these materials which will allow its further correlation with the reaction performance. Therefore, data regarding transmission electron microscopy (TEM), High-resolution transmission electron microscopy (HR-TEM), scanning electron microscopy (SEM) with energy dispersive X-Ray analysis (EDX), X-ray diffraction (XRD) and specific surface area (BET) with pore size distribution (BJH) are provided here. Furtheremore, experimental data on the catalytic activity (in batch and/or dynamic modes) under different reaction conditions (phenol concentration, amine concentration, hydrogen pressure, temperature and alkyl-substituted phenols) are also included in the dataset. The data provided here could support the understanding on the role of active sites nature (Pd or Rh), the effect of operational parameters and the reactivity order for substituted phenols on the aforementioned reaction. Finally, we have included a sample datasheet which could aid the reader to perform preliminary kinetic analysis using the provided dataset.

**Specifications Table**

**Table:** 

Subject	Chemistry: Chemical Engineering, Catalysis
Specific subject area	Conversion of biomass-derived molecules into valuable chemicals by heterogeneous catalysis
Type of data	Tables Figures Images
How the data were acquired	The catalysts were characterized by several techniques: Transmission electron microscopy (TEM) images were taken on a JOEL JEM 1200 EXII microscope at 120 kV to inspect particle size distribution and derive metal dispersion. The morphology and plane dimensions of the metal clusters were inspected from high-resolution transmission electron microscopy (HRTEM) images and electron diffraction patterns ana obtained from a JEOL JEM-2200FS microscope (JEOL, Mitaka, Tokyo) with double aberration correction, operated at an accelerating voltage of 200 kV. The composition was estimated from scanning electron microscopy coupled to energy-dispersive X-ray spectroscopy (SEM-EDX) analysis in a Hitachi SU3500 microscope at 20 kV. The crystalline phases of the catalysts were analysed by measuring the X-ray diffraction (XRD) patterns and comparing the results to standards in a Database. The textural characterization of the catalyst was performed in a Micromeritics 3-Flex instrument by applying BET and BHJ models to the N_2_ physisorption isotherms. The liquid phase reaction was carried out in 4 mL and 20mL autoclave reactors equipped with a gas line for pressurization (H_2_) and controls for temperature and stirring speed. Chemical identities were analysed in a gas chromatograph (Clarus 690, Perkin Elmer) equipped with a mass spectrometer (QS8). Quantification of the products was carried out in a gas chromatograph (SRI, Model 8610) equipped with an FID detector.
Data format	Raw Tables Figures
Description of data collection	The catalytic experiments were carried out for phenolics in autoclave reactors (4 mL and 20 mL), equipped with temperature, pressure and stirring controls. The reaction was performed between 80 and 160°C, 0 – 2.5 bar of H_2_ and 0 – 0.40 mol/L C°_CyA_. Prior to characterizations and reactions, the catalysts (Rh/C and Pd/C) were thermally treated under a constant H_2_ flow.
Data source location	• Institution: Universidad del Bío-Bío • City/Town/Region: Concepción/Concepción/Región del Biobío • Country: Chile
Data accessibility	Repository name: Dataset on the characterization, and application of Pd and Rh-based catalysts in the reductive amination of biomass-derived phenolics **Data identification number:**http://dx.doi.org/10.17632/z5bypjm62y.1 **Direct URL to data:**https://data.mendeley.com/datasets/z5bypjm62y/1

## Value of the Data

• Biomass-derived phenols have significant potential to be used as a molecular platform for amine synthesis. The data presented here includes details on the characterization and activity results for two of the most widespread used catalysts (Pd/C and Rh/C). Therefore, it could support the understanding and elaboration of theories on the role of the active sites in the mentioned reactions.
• The provided dataset can be used to establish correlations between structural properties of catalysts (metal site, cluster size and textural properties) and reaction conditions with the reaction pathways describing the phenolics reductive amination. Thus, it could support the understanding of the reaction performance and the decision-making during process design.
• The dataset from kinetic measurements of the phenols reductive amination is essential for researchers working in the field of green chemistry and industrials seeking for alternatives in the production of amines.

## 1 Data Description

### 1.1 Catalyst characterization

The data referenced here are shared as a MendeleyData repository to facilitate access and sharing of information [5]. This includes raw (*.xls and *.TIFF) files with (i) TEM and HR-TEM images, spreadsheets with the calculation of the particle size distribution and their respective histograms and (ii) XRD analyses performed on Pd/C and Rh/C. In addition, there are pdf files with (iii) SEM-EDS images and the corresponding quantitative analysis of all the elements that compose the catalysts and, (iv) the reports of the surface area, pore volume and pore size analysis of the Pd/C and Rh/C surface.

### 1.2 Catalytic assays

Table 1 and Table 2 presents the conversion and selectivity to the products generated by the liquid phase reaction of phenol with cyclohexylamine over Pd/C and Rh/C catalysts under different reaction conditions. Raw data can be accessed at a Mendeley data repository [5].

**Table 1:** Conversions of reactants generated by the reductive amination of phenol on Pd/C under different reaction conditions. C^0^_PhOH_ = 0.2 mol/L, t_R_ = 6 h and 1.5 mL of tert-amyl alcohol as reaction medium.

Parameter	*X_PhOH_	*X_CyA_	⁎⁎S_DCyA_	⁎⁎S_CyO_	⁎⁎S_CyPhA_	⁎⁎S_Imine_
Amine (mol/L)						

0.0	19%	0%	0%	100%	0%	0%
0.15	38%	76%	82%	0%	0%	18%
0.3	45%	44%	99%	0%	0%	1%
0.4	56%	89%	74%	0%	0%	26%

H_2_ pressure (bar)						

0.5	11%	28%	58%	0%	7%	35%
1.0	45%	44%	99%	0%	0%	1%
1.5	10%	44%	91%	0%	2%	7%

Temperature (°C)						

80	12%	0%	92%	0%	0%	8%
100	34%	8%	100%	0%	0%	0%
120	45%	44%	99%	0%	0%	1%
140	54%	64%	95%	0%	4%	1%

⁎ Xi = conversion of i-th reactant → i = PhOH (Phenol), CyA (cyclohexylamine).

⁎⁎ Si = selectivity of the i-th product→i = DCyA (dicyclohexylamine), CyO (cyclohexanone), CyPhA (cyclohexylaniline), Imine (N-cyclohexaneimine).

**Table 2:** Conversions of reactants generated by the reductive amination of phenol on Rh/C under different reaction conditions. C^0^_PhOH_ = 0.2 mol/L, t_R_ = 6 h and 1.5 mL of tert-amyl alcohol as reaction medium.

	Conversions		Selectivity			
Parameter	X_PhOH_	X_CyA_	S_DCyA_	S_CyO_	S_CyPhA_	S_Imine_
Amine (mol/L)						

0.0	100%	0%	0%	100%	0%	0%
0.15	100%	84%	65%	34%	0%	1%
0.3	100%	72%	72%	24%	1%	3%
0.4	100%	65%	83%	3%	0%	14%

H_2_ pressure (bar)						

0.5	20%	48%	65%	6%	0%	29%
1.0	100%	72%	72%	24%	0%	4%
1.5	100%	88%	93%	6%	0%	1%

Temperature (°C)						

80	100%	48%	93%	7%	0%	0%
100	100%	67%	96%	4%	0%	0%
120	100%	72%	72%	24%	0%	4%
140	100%	76%	94%	5%	0%	1%

* Xi = conversion of i-th reactant → i = PhOH (Phenol), CyA (cyclohexylamine).

^⁎⁎^Si = selectivity of the i-th product→i = DCyA (dicyclohexylamine), CyO (cyclohexanone), CyPhA (cyclohexylaniline), Imine (N-cyclohexaneimine).

Tables 3 to 6 present the temporal measurements of the tests under batch-reactor conditions on Pd/C and Tables 7 to 10 the same results but for measurements on Rh/C. Each table shows the phenol conversion with respect to different concentrations of CyA, PhOH, H_2_ and various reaction temperatures. Raw data can be accessed at a Mendeley data repository [5].

**Table 3:** Phenol conversion results using different initial concentrations of CyA over Pd/C [T = 393 K, P_H2_= 1.5 bar and C^0^_PhOH_ = 0.2 mol/L]. m_cat_ = 0.3 g, V_SOLV_ = 15 mL (TAA).

Effect of cyclohexylamine concentration				
Time (min)	X_PhOH_ C°_CyA_=0.1mol/L	X_PhOH_ C°_CyA_=0.2mol/L	X_PhOH_ C°_CyA_=0.3mol/L	X_PhOH_ C°_CyA_=0.4mol/L
0	0%	0%	0%	0%
30	25%	18%	24%	22%
60	32%	39%	29%	30%
90	39%	52%	51%	71%
150	66%	83%	77%	87%
210	80%	89%	88%	95%
270	88%	94%	95%	100%
330	94%	95%	96%	100%

* X_PhOH_ = Phenol conversion.

**Table 4:** Phenol conversion results using different hydrogen pressures over Pd/C [T = 393 K, C^0^_PhOH_ = 0.2 mol/L and C°_CyA_ = 0.3 mol/L]. m_cat_ = 0.3 g, V_SOLV_ = 15 mL (TAA).

Effect of hydrogen pressure			
Time (min)	X_PhOH_ p^0^_H2_ = 1.0 bar	X_PhOH_ p^0^_H2_ = 1.5 bar	X_PhOH_ p^0^_H2_ = 2.5 bar
0	0%	0%	0%
30	6%	24%	13%
60	7%	29%	19%
90	25%	51%	34%
150	48%	77%	72%
210	67%	88%	92%
270	80%	95%	97%
330	84%	96%	98%

* X_PhOH_ = Phenol conversion.

**Table 5:** Phenol conversion results using different initial concentrations of PhOH over Pd/C [T = 393 K, P_H2_= 1.5 bar and C°_CyA_ = 0.3 mol/L]. m_cat_ = 0.3 g, V_SOLV_ = 15 mL (TAA).

Effect of phenol concentration			
Time (min)	X_PhOH_ C^0^_PhOH_=0.1mol/L	X_PhOH_ C^0^_PhOH_=0.2mol/L	X_PhOH_ C^0^_PhOH_=0.3mol/L
0	0%	0%	0%
30	6%	15%	20%
60	18%	21%	39%
90	27%	44%	50%
150	48%	63%	61%
210	68%	75%	78%
270	78%	87%	84%
330	89%	93%	93%

* X_PhOH_ = Phenol conversion.

**Table 6:** Phenol conversion results using different temperature over Pd/C [P_H2_= 1.5 bar, C^0^_PhOH_ = 0.2 and C°_CyA_ = 0.3 mol/L]. m_cat_ = 0.3 g, V_SOLV_ = 15 mL (TAA).

Effect of temperature			
Time (min)	X_PhOH_ T = 373 K	X_PhOH_ T = 393 K	X_PhOH_ T = 413 K
0	0%	0%	0%
30	0.8%	24%	22%
60	1%	29%	34%
90	2%	51%	62%
150	15%	77%	89%
210	22%	88%	97%
270	23%	95%	99%
330	26%	98%	100%

* X_PhOH_ = Phenol conversion.

**Table 7:** Phenol conversion results using different initial concentrations of CyA over Rh/C [T = 393 K, P_H2_= 1.5 bar and C^0^_PhOH_ = 0.2 mol/L]. m_cat_ = 0.3 g, V_SOLV_ = 15 mL (TAA).

Effect of cyclohexylamine concentration				
Time (min)	X_PhOH_ C°_CyA_=0.1mol/L	X_PhOH_ C°_CyA_=0.2mol/L	X_PhOH_ C°_CyA_=0.3mol/L	X_PhOH_ C°_CyA_=0.4mol/L
0	0%	0%	0%	0%
15	9%	34%	15%	7%
30	18%	71%	58%	14%
45	30%	85%	70%	21%
60	41%	92%	73%	38%
90	60%	99%	86%	92%
120	72%	100%	100%	100%
180	87%	100%	100%	100%

* X_PhOH_ = Phenol conversion.

**Table 8:** Phenol conversion results using different hydrogen pressures over Rh/C [T = 393 K, C^0^_PhOH_ = 0.2 mol/L and C°_CyA_ = 0.3 mol/L]. m_cat_ = 0.3 g, V_SOLV_ = 15 mL (TAA).

Effect of hydrogen pressure			
Time (min)	X_PhOH_ p^0^_H2_ = 1.0 bar	X_PhOH_ p^0^_H2_ = 1.5 bar	X_PhOH_ p^0^_H2_ = 2.5 bar
0	0%	0%	0%
15	20%	15%	20%
30	34%	58%	59%
45	45%	71%	69%
60	48%	74%	89%
90	58%	86%	90%
120	89%	100%	99%
180	100%	100%	100%

* X_PhOH_ = Phenol conversion.

**Table 9:** Phenol conversion results using different initial concentrations of PhOH over Rh/C [T = 393 K, P_H2_= 1.5 bar and C°_CyA_ = 0.3 mol/L]. m_cat_ = 0.3 g, V_SOLV_ = 15 mL (TAA).

Effect of phenol concentration			
Time (min)	X_PhOH_ C^0^_PhOH_=0.1mol/L	X_PhOH_ C^0^_PhOH_=0.2mol/L	X_PhOH_ C^0^_PhOH_=0.3mol/L
0	0%	0%	0%
15	59%	15%	7%
30	64%	58%	13%
45	64%	70%	32%
60	69%	73%	43%
90	90%	86%	86%
120	100%	99%	100%
180	100%	100%	100%

* X_PhOH_ = Phenol conversion.

**Table 10:** Phenol conversion results using different temperature over Rh/C [P_H2_= 1.5 bar, C^0^_PhOH_ = 0.2 and C°_CyA_ = 0.3 mol/L]. m_cat_ = 0.3 g, V_SOLV_ = 15 mL (TAA).

Effect of temperature			
Time (min)	X_PhOH_ T = 373 K	X_PhOH_ T = 393 K	X_PhOH_ T = 413 K
0	0%	0%	0%
15	16%	15%	17%
30	44%	58%	38%
45	54%	71%	62%
60	67%	74%	79%
90	78%	83%	87%
120	90%	100%	99%
180	100%	100%	100%

* X_PhOH_ = Phenol conversion.

**Fig. 1:**
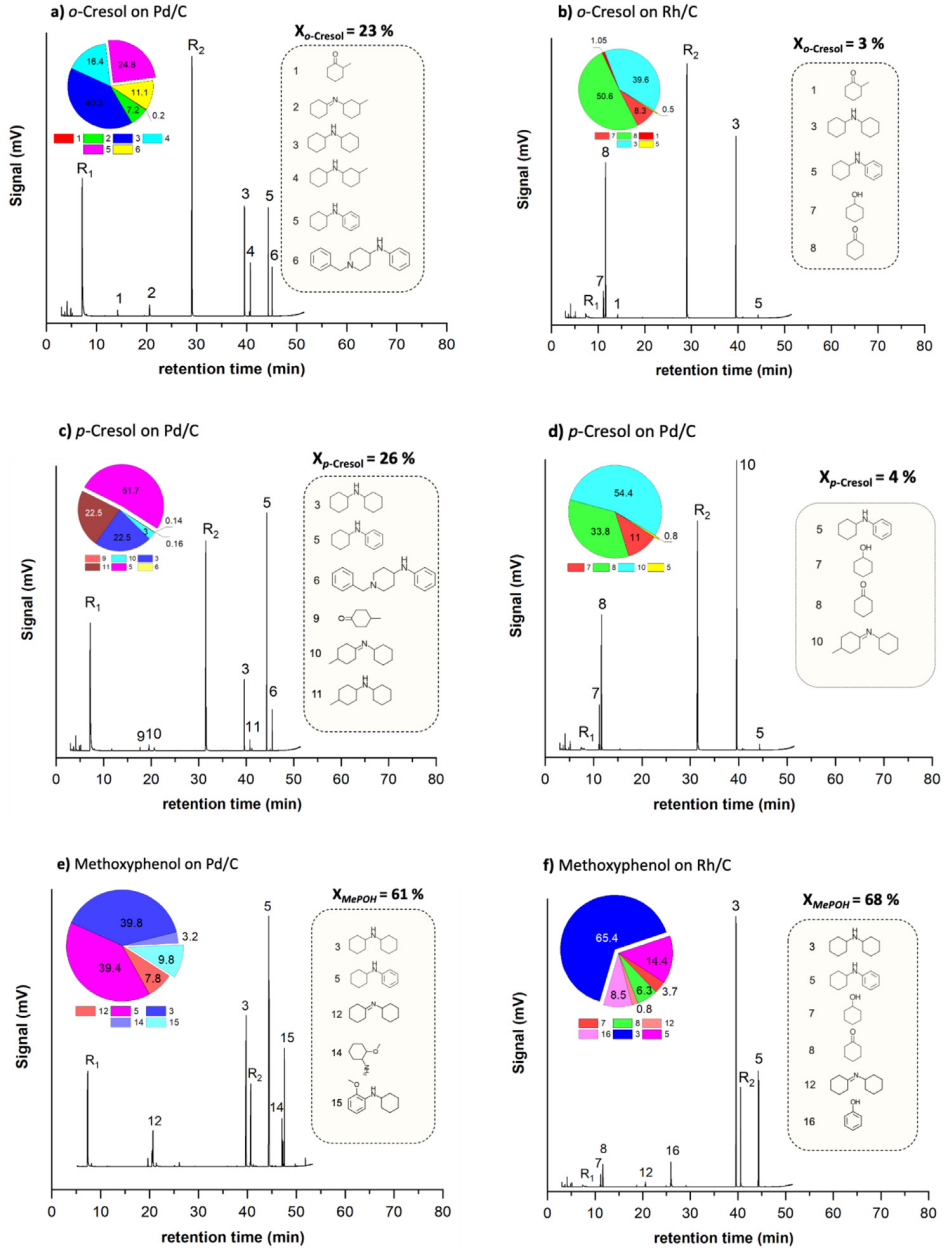
Product distribution of the activity test using lignin-model phenolics for reductive amination with cyclohexylamine on Pd/C. a) o-cresol on Pd/C, b) o-cresol on Rh/C, c) p-cresol on Pd/C d) p-cresol on Rh/C and e) Methoxyphenol on Pd/C f) Methoxyphenol on Rh/C. Reaction conditions: C^0^_Phenolics_ = 0.2 mol/L, C°_CyA_ = 0.3 mol/L, P_H2_= 1.5 bar, t_R_ = 6 h and 1.5 mL of tert-amyl alcohol as reaction medium at 393 K.

**Fig. 2:**
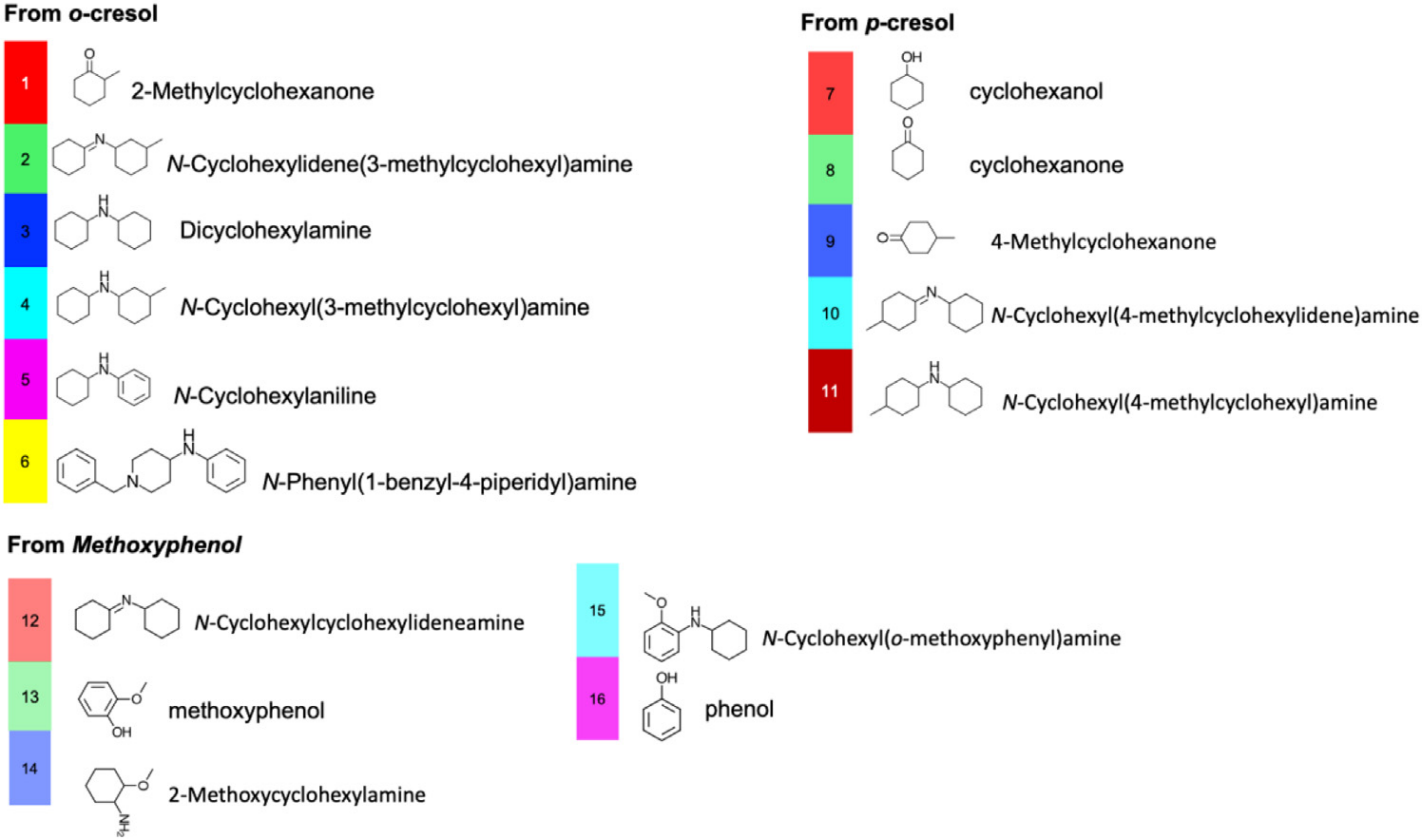
Colour code and IUPAC names of all the structures in Figs. 1(a) to 1(f).

Figs. 1(a) to 1(f) present the GC-MS chromatogram and the product distribution (area-related selectivity) generated by the liquid-phase batch reaction of different lignin-model phenolics with cyclohexylamine over Pd/C and Rh/C catalysts, respectively. Moreover, Fig. 2 represent the colour code and the IUPAC names of all the structures in these figures. The raw data for these experiments can be accessed in a Mendeley data repository [5].

## 2 Experimental Design, Materials and Methods

**Material:** Commercial 10% Pd/C (CAS-87104) and 5% Rh/C (CAS-7440-16-6) were purchased from Merck Group (Chile). The catalysts were dried and then sieved to obtain particle sizes<75μm to ensure efficient agitation in the autoclave reactors. In order to obtain zero-valent Pd and Rh sites, the catalysts were pre-treated at 400 °C (heating rate of 1.5°C/min) for 2 h under a stream of 40 mL/min H_2_ (99.999%, Airliquide, Chile) in a U-shaped fixed-bed reactor (118 mm ID), prior to use.

**Characterization of catalysts:** The morphology and size of the metal clusters were inspected by transmission electron microscopy (TEM) using a JEOL JEM 1200 EXII microscope (Jeol, Peabody, Massachusetts, USA) at 120 kV. The catalysts were dispersed in ethanol and placed on a carbon-coated copper grid used as substrate. The average particle sizes of the catalysts were obtained using the methodology proposed by Vannice [6] through the values determined from TEM images processed with ImageJ software.

(1) $$dp_{i}=\frac{\sum_{j=1}^{n}n_{j}\times d_{\text{pj}}^{4}}{\sum_{j=1}^{n}n_{j}\times d_{\text{pj}}^{3}}$$

(2) $$\text{dp}=\frac{\sum_{j=1}^{n}n_{i}\times \;d_{\text{pi}}}{\sum_{i=1}^{n}n_{i}}$$

Metal dispersion was determined from the average size of the particles considering hemispherical surface as:

(3) $$D=\frac{6\;\times \left(\text{vm}\;/\;\text{am}\right)}{\text{dp}}$$

Here, **D** is the metal (Pd or Rh) dispersion in the catalyst, **v_m_** is the volume occupied by an atom in bulk metal (Pd = 14.7 Å^3^, Rh = 13.78 Å^3^), **a_m_** is the area occupied by a surface atom (Pd = 7.93 Å^2^, Rh = 7.58 Å^2^), and **dp** is the average cluster size obtained from TEM analysis.

High-resolution transmission electron microscopy (HRTEM) images and electron diffraction pattern images provided more information on the local structure and surface morphology of the metal clusters in the catalysts. These analyses were performed on a JEOL JEM-2200FS microscope (JEOL, Mitaka, Tokyo) with double aberration correction, operated at an accelerating voltage of 200 kV.

Scanning Electron Microscopy (SEM) measurements were carried out on Hitachi SU3500 device operated at an accelerating voltage of 20kV and coupled to a Bruker XFlash 610M energy dispersive X-ray spectroscopy (EDX) accessory for semi-quantitative spectral analysis.

The bulk crystalline phases of reduced Pd/C and Rh/C were analyzed from X-ray diffraction (XRD) patterns recorded for 2θ between 3° and 90° at a rate of 0.02 °/s. The analysis was performed on a Bruker D4 diffractometer with CuKα radiation (λ = 0.15418 nm) at 40 kV and 20 mA. The identification of the crystalline phases was carried out by a search-match procedure through Mercury 3.7 software, using the Crystallography Open Database (COD) [7]. In addition, the sizes of the metallic particles were estimated using the Scherrer equation [8].

(4) $$L=\frac{K*\lambda }{\;\beta *\text{cos}\left(\theta \right)}$$

where **L** is the crystallite size in nm, **λ** is the CuKα wavelength in nm**; β** is the full width at half maximum intensity (FWHM) in radians, K is a constant near to unit; and **θ** is the Bragg's angle (rad).

The specific surface area (BET) and pore size distribution (BJH) of the catalyst were determined by N_2_ physisorption at 77 K on a Micromeritics 3-Flex instrument. The samples (∼100 mg) were degassed for 4 h in vacuum at 150°C and under a flow of N_2_ gas prior to analysis. The Brunauer-Emmett-Teller (BET) model was used to determine the specific surface area under the relative pressure range of 0.005–0.899. The average pore diameter (Dp) was estimated from the BJH model applied on the adsorption isotherms.

### 2.1 Catalytic assays

Liquid-phase reactions of phenols such as, phenol (CAS-108-95-2, >99%), o-cresol (CAS-95-48-7, >99%), p-cresol (CAS-106-44-5, >98%), methoxyphenol (CAS-90-05-1, >98%) with cyclohexylamine (CAS-108-91-8, >99%) in tert-amyl alcohol (CAS-75-85-4, >99%) under H_2_ (99.999%, Airliquide, Chile) atmosphere were carried out according to the experimental designs depicted in Tables 11, 12 and 13.

**Table 11:** Batch reaction conditions for the amination of phenol with cyclohexylamine. Solvent = Tert-Amyl alcohol, and Pd/C (10 wt.%) or Rh/C (5 wt.%) as catalytic material.

Run	Cyclohexylamine (mol/L)	H_2_(g) (bar)	Catalyst^a^ (mol%)	Temperature (K)	Solvent (1.5 mL)	Phenol (mol/L)
1	0	1	6	393	TAA	0.2
2	0.15	1	6	393	TAA	0.2
3	0.3	1	6	393	TAA	0.2
4	0.4	1	6	393	TAA	0.2
5	0.3	0.5	6	393	TAA	0.2
6	0.3	1.5	6	393	TAA	0.2
7	0.3	1	6	353	TAA	0.2
8	0.3	1	6	373	TAA	0.2
9	0.3	1	6	413	TAA	0.2

a Referred to 6 mol% equivalent of Pd or Rh with respect to the substrate (phenol).

**Table 12:** Dynamic reaction conditions for the amination of phenol with cyclohexylamine. Solvent = Tert-Amyl alcohol, and Pd/C (10 wt.%) or Rh/C (5 wt.%) as catalytic material.

Run	Cyclohexylamine (mol/L)	H_2_(g) (bar)	Catalyst^a^ (mol%)	Temperature (K)	Solvent (15 mL)	Phenol (mol/L)
1	0.1	1.5	6	393	TAA	0.2
2	0.2	1.5	6	393	TAA	0.2
3	0.3	1.5	6	393	TAA	0.2
4	0.4	1.5	6	393	TAA	0.2
5	0.3	1	6	393	TAA	0.2
6	0.3	2.5	6	393	TAA	0.2
7	0.3	1.5	6	393	TAA	0.1
8	0.3	1.5	6	393	TAA	0.3
9	0.3	1.5	6	373	TAA	0.2
10	0.3	1.5	6	413	TAA	0.2

a Referred to 6 mol% equivalent of Pd or Rh with respect to the substrate (phenol).

**Table 13:** Batch reaction conditions for the amination of different phenols with cyclohexylamine. Solvent = Tert-Amyl alcohol, and Pd/C (10 wt.%) or Rh/C (5 wt.%) as catalytic material.

Run	Cyclohexylamine (mol/L)	H_2_(g) (bar)	Catalyst^a^ (mol%)	Temperature (K)	Solvent (1.5 mL)	Phenols (0.2 mol/L)
1	0.3	1	6	393	TAA	o-Cresol
2	0.3	1	6	393	TAA	p-Cresol
3	0.3	1	6	393	TAA	Metoxyphenol

a Referred to 6 mol% equivalent of Pd or Rh with respect to the substrate (phenols).

**Fig. 3:**
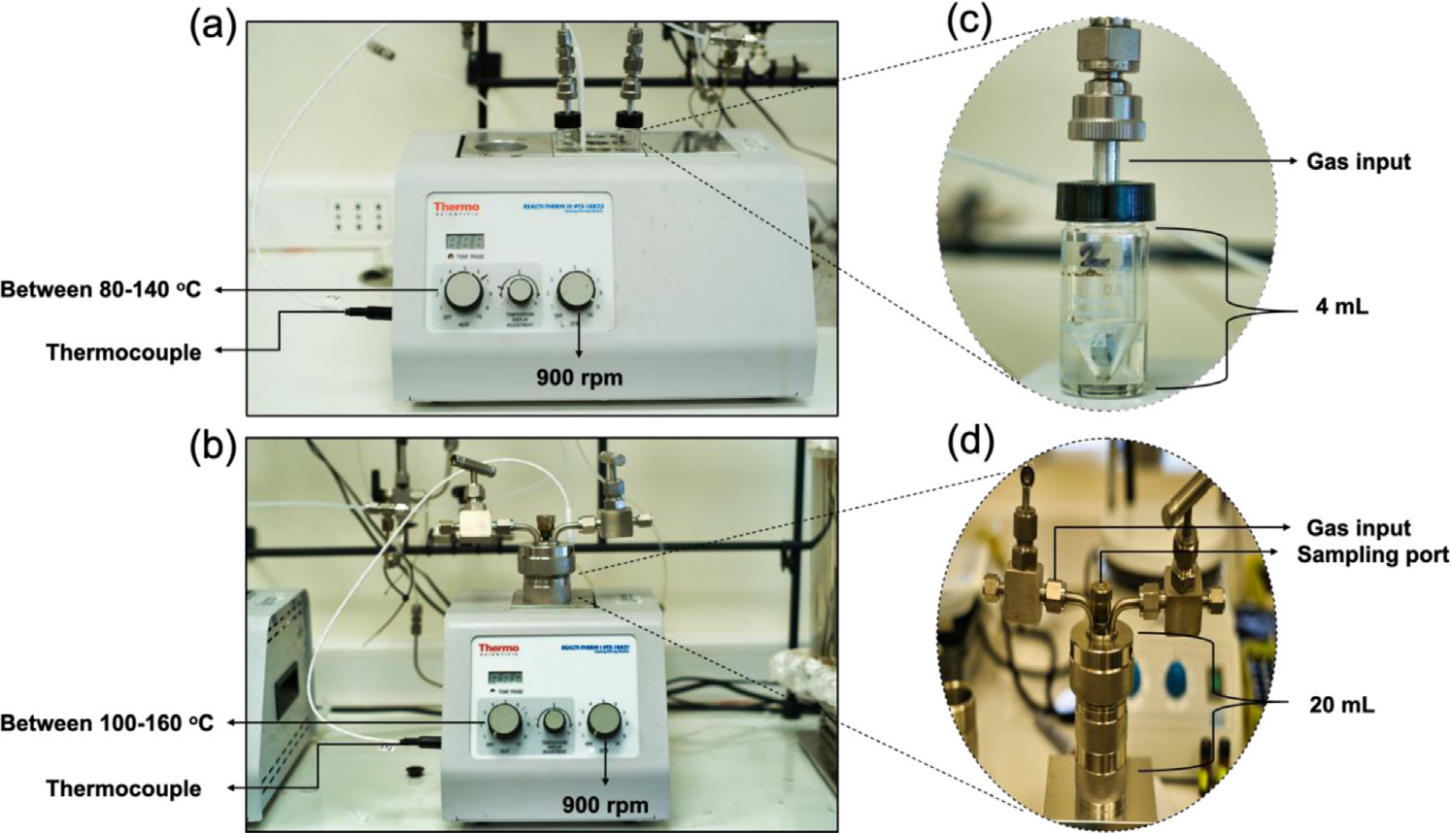
Reaction systems used for reactivity and kinetics measurements under (a) batch and (b) dynamic conditions with (c) glass autoclave and (d) SS316 reactors.

The study under **batch reaction** conditions (T, PH_2_, C^0^_i_) was performed in 4 mL reinforced glass autoclave reactors, and the **dynamic kinetic measurements** were carried out under similar reaction conditions in a 20 mL SS316 reactor equipped with a sampling port (Fig. 3).

Procedure for **batch and dynamic reactions**: The reaction mixture was prepared in a PTFE liner of an autoclave reactor (glass autoclave reactors or SS316 reactor). The liner was double washed (acid and deionized water) and dried prior to the experiments. The reactor was placed on a precision balance (Biobase BA504B, ±0.1 mg) to add the phenolics with an SS316 spatula. Then, the amine (cyclohexylamine) and solvent (tert-amyl alcohol) were loaded into the autoclave using an automatic micropipette (Nichipet EXII, 10 - 100μL and 100 - 1000μL) at the specific volumes, to ensure the concentration of the corresponding experiment (0 – 0.40 mol/L). Finally, a Teflon-coated magnetic stirrer and the corresponding mass of catalyst (Pd/C or Rh/C) were introduced into the reactor. The stability of the catalysts allowed them to be handled without special precautions.

Leaks tests were performed with inert He (99.995%, Airliquide, Chile) at 2 bar to ensure hermetic seal of the reactor. The He flow in the system was provided by a mass flow controller (Aalborg MFC 17007). The reactor was purged three times to 1 bar He (99.995%, Airliquide, Chile), and subsequently the total pressure (P_He_+P_H2_) was fixed to maintain 2 bar during the reaction under Batch conditions (hydrogen pressures were varied between 0.5 bar to 1.5 bar). In the case of the dynamic tests, the effect of H_2_ pressure was varied to obtain H_2_ concentrations between 1 bar to 2.5 bar. The H_2_ flow in the system was provided by a mass flow controller (Aalborg MFC 17007). The reactors were placed in a Reacti-ThermTM system (Thermofisher, USA), equipped with an external temperature probe and magnetic stirring (Fig. 3 (a) to (d)). After pressurizing the system, the reactor was heated up to the reaction temperature (reaction temperature was studied between 80 and 160 °C) and stirring was fixed at 900 r.p.m. In both, the batch and kinetic experiments, the reaction time was fixed at 6 hours. The continuous sampling of reaction products was done via port equipped with a GC-Septa using a 100 µL Hamilton syringe, and the content was transferred to 2 mL Amber glass vials using a Teflon filter (0.45 μm). Finally, the samples were stored at -40 °C until analysis.

The reaction products were identified on a gas chromatograph (Clarus 690, Perkin Elmer) equipped with a quadrupole mass detector (SQ8S, Perkin Elmer). Compounds were separated on an Elite 1701 column (30 m × 0.25 mm × 0.25 μm) using He as carrier gas at 15 mL min^−1^ and a heating ramp from 45 to 280°C at 2.5°C min^−1^. The compounds were ionized and the resulting mass spectra were compared to the NIST library standard spectra database over an m/z range of 30-600 Da. Due to the significant number of compounds (16 products) generated from the amination of substituted phenols, their selectivity were determined through the peak areas identified in mass spectra.

All samples related to the phenol amination reaction were analysed by ex situ gas chromatography on an SRI chromatograph (model 8610) equipped with a flame ionization detector (FID) and an MTX-5 column (30 m X 0.25 mm X 0.1 μm). Quantification of the products was done using nonane (CAS-111-84-2, >99%) as internal standard, and calibration curves for commercial reagents [Imine (N-cyclohexaneimine, CAS-1132-38-3, >95%;), cyclohexanone (CAS-108-94-1, >99%), Dicyclohexylamine CAS-101-83-7, >99%)], Cyclohexylaniline (CAS-1821-36-9, >98%)].

GC analysis allowed the evaluation of the reaction yield through several quantitative parameters:

The conversion at reaction time t, defined as:

(5) $$X_{i}=\left(n_{i,o}-n_{i,t}\right)\;/\;n_{i,o},\;i=\text{reactants}$$

The TOF was defined as described by Guo et al.[9].

(6) $$\text{TOF}=\frac{X_{i}\times \left(n_{i,o}\right)\;/\;n_{\text{metal}}}{t\left(h\right)\;\times D_{i}},\;i=\text{reactants}$$

Then, the product selectivity was computed as suggested by Vannice [6]

(7) $$S_{i}=\frac{r_{i}}{\sum_{i}r_{i}}$$

The conditions and details of the analytical methods and procedures for yield and kinetic studies, as well as for product identification and quantification, can be found in a shared MendeleyData repository for easy access and exchange of information [5].

## Ethics Statements

Provided dataset do not involve human subjects nor experiments with animals. Furthermore, it represents original data gathered by the research team thus it does not imply the collection of information from social media or any other public database.

## CRediT authorship contribution statement

**Maray Ortega:** Methodology, Investigation, Data curation. **Benjamin L. Garrido-Lara:** Investigation, Data curation. **Raydel Manrique:** Data curation, Writing – original draft. **Luis E. Arteaga-Pérez:** Conceptualization, Supervision, Writing – review & editing, Funding acquisition.

## Declaration of Competing Interest

The authors declare that they have no known competing financial interests or personal relationships that could have appeared to influence the work reported in this paper.

## Data Availability

Dataset on the characterization, and application of Pd and Rh-based catalysts in the reductive amination of biomass-derived phenolics (Original data) (Mendeley Data).
